# MiRNAs in Systemic Sclerosis Patients with Pulmonary Arterial Hypertension: Markers and Effectors

**DOI:** 10.3390/biomedicines10030629

**Published:** 2022-03-08

**Authors:** Mor Zaaroor Levy, Noa Rabinowicz, Maia Yamila Kohon, Avshalom Shalom, Ariel Berl, Tzipi Hornik-Lurie, Liat Drucker, Shelly Tartakover Matalon, Yair Levy

**Affiliations:** 1 Sackler Faculty of Medicine, Tel Aviv University, Tel Aviv 6997801, Israel; mor.zaaroor@yahoo.com (M.Z.L.); noa_uziel@hotmail.com (N.R.); maia.kohon@clalit.org.il (M.Y.K.); avshalom.shalom@clalit.org.il (A.S.); arielberl23@gmail.com (A.B.); druckerl@clalit.org.il (L.D.); 2 Autoimmune Research Laboratory, Meir Medical Center, Kfar Saba 4428164, Israel; 3 Department of Plastic Surgery, Meir Medical Center, Kfar Saba 4428164, Israel; 4 Research Authority, Meir Medical Center, Kfar Saba 4428164, Israel; tzipi.hornik@clalit.org.il; 5 Oncogenetic Laboratory, Meir Medical Center, Kfar Saba 4428164, Israel; 6 Department of Internal Medicine E, Meir Medical Center, Kfar Saba 4428164, Israel

**Keywords:** systemic sclerosis, pulmonary arterial hypertension (PAH), biomarkers, miRNA, complement, myofibroblasts, macitentan

## Abstract

Background: Pulmonary arterial hypertension (PAH) is a major cause of death in systemic sclerosis (SSc). Early detection may improve patient outcomes. Methods: We searched for circulating miRNAs that would constitute biomarkers in SSc patients with PAH (SSc-PAH). We compared miRNA levels and laboratory parameters while evaluating miRNA levels in white blood cells (WBCs) and myofibroblasts. Results: Our study found: 1) miR-26 and miR-let-7d levels were significantly lower in SSc-PAH (*n* = 12) versus SSc without PAH (SSc-noPAH) patients (*n* = 25); 2) a positive correlation between miR-26 and miR-let-7d and complement-C3; 3) GO-annotations of genes that are miR-26/miR-let-7d targets and that are expressed in myofibroblast cells, suggesting that these miRNAs regulate the TGF-β-pathway; 4) reduced levels of both miRNAs accompanied fibroblast differentiation to myofibroblasts, while macitentan (endothelin receptor-antagonist) increased the levels. WBCs of SSc-noPAH and SSc-PAH patients contained equal amounts of miR-26/miR-let-7d. During the study, an echocardiograph that predicted PAH development, showed increased pulmonary artery pressure in three SSc-noPAH patients. At study initiation, those patients and an additional SSc-noPAH patient, who eventually developed PAH, had miR-let-7d/miR-26 levels similar to those of SSc-PAH patients. This implies that reduced miR-let-7d/miR-26 levels might be an early indication of PAH. Conclusions: miR-26 and miR-let-7d may be serological markers for SSc-PAH. The results of our study suggest their involvement in myofibroblast differentiation and complement pathway activation, both of which are active in PAH development.

## 1. Introduction

Systemic sclerosis (SSc) is a rare connective tissue disease that primarily affects women. Its main manifestations are skin and visceral fibrosis, vascular hyperactivity and musculoskeletal changes [1]. It is generally accepted that an interplay between genetic and environmental factors induce SSc-specific gene programs in several cell populations, including immune cells, endothelial cells and myofibroblasts [2,3]. SSc involves three main pathophysiologic pathways: (1) An autoimmune attack, which is the first marker of the disease, (2) vascular injury followed by defective neovascularization and impaired remodeling (3) and extensive tissue fibrosis [3]. 

Pulmonary arterial hypertension (PAH) is a major cause of death in approximately 12% of SSc patients [3]. Older age at scleroderma onset is a risk factor for PAH [4,5]. SSc-associated PAH (SSc-PAH) is caused by functional alterations and structural fibroproliferative vasculopathy affecting the small- and medium-size pulmonary arteries, leading to an increase in mean pulmonary artery pressure of >20 mmHg in echocardiography (echo) at rest [6,7]. SSc-PAH has a poor prognosis, with a five-year overall survival rate of 50% [8].

Early detection of PAH in patients with SSc is essential because an asymptomatic phase is common and early treatment can significantly improve clinical outcomes and positively affect survival [5]. Accurate diagnosis of PAH is clinically challenging and relies on right heart catheterization. This invasive procedure is not suitable for screening and is typically performed only for patients with a high index of suspicion based on echo and other criteria that are less sensitive [8]. In recent years, great efforts have been directed to finding serum-based diagnostic biomarkers for SSc-PAH that would allow for rapid, noninvasive screening, and early diagnosis. 

As the pathogenesis of SSc is influenced by environmental factors, it is reasonable to assume that they induce epigenetic regulatory changes that alter disease outcomes [9]. RNA interference via microRNAs (miRNA) is a leading mechanism for initiating and maintaining epigenetic changes. Previous publications showed that SSc patients have different levels of certain miRNAs in their circulation and skin fibroblasts compared to healthy individuals [9,10]. Yet, only a few reports described differences in miRNA expression between patients with SSc that do not have PAH (SSc-noPAH) vs. SSc-PAH.

The aim of this study was to find markers that would allow diagnosis of PAH in the early stages. We searched for a marker that would not require significant invasive intervention. Obtaining a blood sample does not require a complex invasive process; therefore, we chose to perform our analysis on blood samples from our biobank. We selected plasma samples of 25 SSc patients without PAH (SSc-noPAH) and 12 SSc-PAH patients. We searched for a molecule that affects signal transmission processes and the development of PAH. As miRNAs are often used to identify pathologic conditions such as cancers and autoimmune diseases, in the current study, we searched for circulating miRNAs that could serve as biomarkers for SSc patients with PAH (SSc-PAH). To learn more about the effects of these miRNAs in PAH, we correlated patients’ miRNA levels and laboratory parameters and performed a bioinformatics analysis to find biological annotations of genes common to the miRNA targets and to the biology of immune, endothelial and myofibroblast cells. Since plasma miRNAs represent the total miRNAs secreted from various cells in the body, we also aimed to determine which cell population(s) is responsible for the changes in plasma miRNAs by evaluating levels of the biomarker miRNAs in white blood cells (WBCs) of SSc-noPAH and SSc-PAH patients, as well as in activated myofibroblasts. 

## 2. Materials and Methods

### 2.1. Patient Data

The current study included the entire population of SSc-PAH patients at Meir Medical Center (12 patients) and 25 SSc-noPAH patients, whose blood samples were consecutively collected. SSc patients were diagnosed according to 2013 EULAR/ACR classification, by using Doppler echocardiography (EPIQCVx Release 5.0.2, PHILIPS)) and right heart catheterization. Doppler echocardiography estimated PASP (pulmonary artery systolic pressure, where PASP = 4 (TRV)2) exceeding 35 to 40 mmHg was considered elevated. When PAH was suspected based on this screening, we proceed to right-heart catheterization (RHC). Cut-off values for establishing SSc-PAH in right heart catheterization (RHC) is mPAP >20 mmHg. All samples were from patients at least 2 years after disease onset. Their clinical features are described in Table 1. The clinical data that were collected for each patient included C3 and C4 fragments of the complement, total IgG, IgG1–4 levels, autoantibodies positivity (anti-centromere, anti-RNA-polymerase III and anti-DNA-topo-isomerase I) and CRP.

#### 2.1.1. C3, C4 and IgG Evaluation

C3, C4 and IgG levels were evaluated with NAS IGG, NAS C3 and NAS C4 kits (Siemens, Marburg, Germany), according to manufacturer’s instructions. Briefly, the proteins in the plasma form immune complexes with specific antibodies, which scatter a beam of light passed through the sample. The intensity of the scattered light is proportional to the concentration of the relevant protein in the sample. The results were evaluated by comparison with standard values of known concentration.

#### 2.1.2. IgG1-4 Evaluation 

IgG1-4 levels were evaluated using Optilite IgG1-4 kits (Optilite, Birmingham, UK). The method for determining the protein concentration is similar to that of C3 (Section 2.1.1), using the Binding Site analyzer (Optilite, Birmingham, UK). 

#### 2.1.3. CRP Evaluation

CRP was evaluated using CRP Latex reagent (Beckman Coulter, Brea, CA, USA) and the Beckman Coulter System. During evaluation, CRP reacts specifically with anti-human CRP antibodies coated on the latex particles to yield insoluble aggregates. The absorbance of these aggregates is proportional to the CRP concentration in the sample.

#### 2.1.4. Evaluation of Autoantibodies

Screening for autoantibodies was done using the BioPlex 2200 ANA Screen Pack (Bio-Rad, Hercules, CA, USA). Detection was done in a multiplex flow immunoassay with the BioPlex 2200 System (Bio-Rad, Hercules, CA, USA).

### 2.2. Biobank

The Autoimmune Laboratory at Meir Medical Center in Israel manages a rheumatologic bio-bank that contains plasma, serum and blood cells collected from rheumatology patients since April 2018. Blood samples are collected from participants who previously consented and authorized the use of their samples for research purposes. Blood samples were separated into serum, plasma and cells a few minutes after blood draw and immediately placed in a −80 °C freezer.

### 2.3. Blood Collection and Separation of Plasma and WBCs

Blood samples were taken from participants who consented to donate blood. Twenty ml of blood was drawn into a tube with EDTA and centrifuged (3000× *g*, 10 min, room temperature). The plasma was collected into a tube and immediately frozen at −80 °C. Then, the WBCs cells that lay over the RBC along with the top layer of the RBC were collected into a separate test tube and immediately frozen at −80 °C. Prior to RNA extraction, the cells were incubated for 10 min at room temperature with a buffer lysis (Lysing Solution (X10), Beckman Coulter, Villepinte, France), diluted with molecular biology grade water, Dnase and RNase free (Satorius, Beit Haemek, Israel) and centrifuged (1400 rpm, 5 min). The top fluid was discarded and RNA extraction was performed on the remaining cells at the bottom of the test tubes.

### 2.4. RNA Extraction and RT cDNA Synthesis

RNA was extracted from the SSc-noPAH and SSc-PAH patients’ plasma using miRNeasy Serum/Plasma Advanced Kit (QIAGEN, Hilden, Germany) and from the WBCs using miRNeasy Tissue/Cells Advanced mini-Kit (QIAGEN, Hilden, Germany) [11]. Extracted RNA was converted to cDNA using TaqMan Advanced miRNA cDNA Synthesis Kit (Thermo Fisher, Waltham, MA, USA) according to manufacturer’s instructions.

### 2.5. Real-Time Quantitative PCR

PCR reaction was done using TaqMan Fast Advanced Master Mix (Applied Biosystems, Vilnius, Lithuania) and TaqMan Advanced miRNA Assay (Applied Biosystems, Vilnius, Lithuania) for miRNAs hsa-26a-5p, hsa-21-5p, hsa-155-5p, hsa-miR-let-7d-5p, hsa-29a-5p, hsa-145-5p, hsa-150-5p and hsa-23a-5p according to the manufacturer’s instructions (Applied Biosystems, Vilnius, Lithuania). Hsa-16-5p served for normalization of miRNA RT-qPCR expression analysis [11,12].

### 2.6. Isolation of Dermal Fibroblasts

Cells were isolated from the skin of healthy subjects who underwent abdominoplasty in the Plastic Surgery Unit of Meir Medical Center and who provided signed informed consent. The skin was cleaned with 70% ethanol, layered in PBS-penicillin streptomycin 1% and the fat was removed using scissors. Biopsy was cut into 25–30 small pieces (~4 mm), using a Uni Punch (Premier Company, Mumbai, Maharashtra, India), placed in a tube with 7 mL collagenase mix (0.02 g collagenase type 2 (Gibco, New York, NY, USA), 0.001 g DNase (Roche, Mannheim, Germany), 2 mL Trypsin X10 (Biological Industries, Beit Haemek, Israel), 0.1 gr bovine serum albumin (Sigma-Aldrich, St. Louis, MO, USA), in 20 mL Dulbecco’s modified eagle medium (DMEM; Biological Industries, Beit Haemek, Israel) vortexed for 30 s, cultured in an incubator containing a rotating device for 45 min, at 37 °C, shaken and incubated for an additional 45 min. Following incubation, 7 mL of full DMEM (DMEM supplemented with 10% fetal bovine serum (FBS), 2 mM l-glutamine and antibiotics (100 U/mL penicillin, 100 µg/mL streptomycin, nystatin), all from Biological Industries, Beit Haemek, Israel) was added to each test tube and centrifuged (1700 rpm for 8 min). The top fluid was removed and the remaining fluid was cultured in plates with full medium containing 20% FBS in a CO2 incubator at 37 °C. Cells were observed every 2 days and new 20% FBS medium was added. After 4 days, the pieces were discarded and when cells reached 50% confluence, media were replaced with 10% full DMEM.

### 2.7. Cell Culture

Dermal fibroblasts were cultured in full DMEM. Cells were incubated at 37 °C in 5% CO2 and split twice a week using trypsin, upon reaching 80–90% confluence. Cells were used in the experiments in passages 4–9.

### 2.8. Exposure of Dermal Fibroblasts to TGF-β and to Macitentan

Cells were cultured in 96- or 24-well plates with full DMEM that contained 1% FBS. Twenty-four hours later, new medium (control) or TGF-β (10 ng/mL, Peprotech, East Windsor, NJ, USA), with or without macitentan (1) (1 µM) Selleck chemicals, Houston, TX, USA) or DMSO (Sigma-Aldrich, St. Louis, MO, USA) in the same concentration as control (1:50,000), were added to the cells for 48 h or 72 h. Then, the following procedures were performed: (1) Cells were harvested and counted using an automatic cell counter (NanoEntek, Waltham, MA, USA) and trypan-blue (Gibco, New York, NY, USA (72 h); (2) proteins were isolated from the cells for evaluation of αSMA and collagen I levels (western blot, 48 h) and stored at −80 °C; (3) RNA was isolated from the cells for future miRNA analysis (48 h).

### 2.9. Cell Count

Trypan blue (Biological Industries, Beit Haemek, Israel) was mixed in a 1:1 ratio with the cells. Then the cells were counted using automatic cell counter (NanoEntek, Waltham, MA, USA). Live cells remained unstained, while dead cells assimilated the dye. The cell counter counted the number of dyed and undyed cells in each sample. Using these values, it calculated the percentages of dead cells in the culture.

### 2.10. Protein Extraction

Dermal fibroblasts were lysed in a lysis buffer (50 mM Hepes, 150 mM NaCl, 1% Triton X-100, 0.1% SDS, 50 mM NaF, 10 mM NaPPi, 2 mM NaVO_3_, 10 mM EDTA, 2 mM EGTA, 1 mM PMSF and 10 µg/mL Leu-peptin) for 10 min on ice, and centrifuged (15 min, 12,000 RPM, 4 °C). Protein levels were determined by using Pierce BCA protein assay kit (Pierce, Rockford, IL, USA), according to manufacturer’s instructions.

### 2.11. Western Blotting

To assess the levels of αSMA and collagen I in the fibroblasts, protein lysates were mixed (1:5) with sample buffer (250 mM Tris-HCl, pH 6.8, 400 mM DTT, 140 mM SDS, 60% glycerin, 0.02% bromophenol blue) and denatured for 10 min at 65 °C. Proteins (25 µg) from each sample were separated by electrophoresis on SDS-PAGE and wet transferred to a PVDF membrane. Transfer efficiency was validated with Ponceau staining (Sigma-Aldrich, St. Louis, MO, USA). After blocking the non-specific binding sites with 5% milk powder (Difco, Saint-Ferreol, France) in Tris Buffer Saline containing 0.1% Tween (TBS-T) (Milipore Sigma, Molsheim, France), the membranes were incubated with the primary antibodies at 4 °C overnight. We used the following antibodies: Mouse anti-human alpha smooth muscle actin (αSMA, IgG2a, clone: 1A4, ab7817; Abcam, Cambridge, England), rabbit anti-human collagen type I, (IgG, ab34710, Abcam) and rabbit mouse anti-tubulin (clone: B-5-1-2, T516, Sigma Aldrich, St. Louis, MO, USA). Primary antibody was rinsed with TBS-T and TBS (Milipore Sigma)). Bound antibodies were visualized using peroxidase-conjugated secondary antibody goat anti-mouse IgG and goat anti-rabbit IgG (Jackson ImmunoResearch Laboratories, West Grove, PA, USA), followed by enhanced chemiluminescence HRP substrate detection (Milipore Sigma, Waltham, MA, USA). Optical densities were visualized and measured as arbitrary units using an LAS-3000 Imager (Fujifilm, Tokyo, Japan). Results were normalized to tubulin using a multi-gauge V3.0 program (Fujifilm).

### 2.12. Bioinformatics

Targets of the miRNA were found by using 2 software packages (targetscan, http://www.targetscan.org, accessed on 30 October 2021 and mirbd, http://mirdb.org, accessed on 30 October 2021).

Specific sites for miRNA let7d:

http://www.targetscan.org/cgi-bin/targetscan/vert_71/targetscan.cgi?mirg=hsa-miR-let-7d-5p (accessed on 30 October 2021)

http://mirdb.org/cgi-bin/search.cgi?searchType=miRNA&full=mirbase&searchBox=MIMAT0000065 (accessed on 30 October 2021)

Specific sites for miR-26:

http://mirdb.org/cgi-bin/search.cgi?searchType=miRNA&full=mirbase&searchBox=MIMAT0000082 (accessed on 30 October 2021)

http://www.targetscan.org/cgi-bin/targetscan/vert_71/targetscan.cgi?mirg=hsa-miR-26a-5p (accessed on 30 October 2021)

A list of genes involved in (1) endothelial cell biology, (2) myofibroblast cell biology and (3) immune system biology was made by using these websites:

Specific sites for endothelial cell biology:

http://ezbiosystems.com/servicescontent.asp?d_id=174 (accessed on 30 October 2021)

https://www.sciencellonline.com/PS/GK019.pdf (accessed on 30 October 2021)

Specific sites for myofibroblast cell biology:

https://maayanlab.cloud/Harmonizome/gene_set/myofibroblast/TISSUES+Text-mining+Tissue+Protein+Expression+Evidence+Scores (accessed on 30 October 2021)

https://maayanlab.cloud/Harmonizome/gene_set/myofibroblast/GeneRIF+Biological+Term+Annotations (accessed on 30 October 2021)

Specific sites for Immune system biology

https://www.sciencellonline.com/PS/GK072.pdf (accessed on 30 October 2021)

https://www.sciencellonline.com/PS/GK039.pdf (accessed on 30 October 2021)

https://www.thermofisher.com/order/catalog/product/4370573#/4370573 (accessed on 30 October 2021)

Then, Venny software: https://bioinfogp.cnb.csic.es/tools/venny/, (accessed on 30 October 2021) was used to create 6 lists of genes which are common to the miRNA targets and the 3 types of cells. Toppgene software https://toppgene.cchmc.org/navigation/database.jsp, (accessed on 30 October 2021) was used to find annotations for the gene lists.

### 2.13. Statistical Analysis

Demographic, clinical and laboratory characteristics of the patients were presented as medians and percentages, each when appropriate. Due to the small number of patients, the median and 25–75 percentile was used to demonstrate the distribution of non-categorical variables. Percentages were used for categorical variables. Mann–Whitney U tests were used to compare differences between the two independent groups (SSc-noPAH and SSC-PAH) for the continuous, dependent variables. Chi-square tests were used to test relationships between categorical variables and the two independent groups. Spearman’s Rho correlations were used to evaluate the association between the research variables. A partial Spearman’s Rho correlation was used to evaluate the association between the study variables, adjusted for confounding variables. Cell culture experiments with dermal fibroblasts were conducted 4 times and miRNAs in WBCs were evaluated 7 to 8 times. Paired student t-tests were applied to analyze differences between paired data. All statistical analyses were performed using SPSS-PC statistical software, version 27.0 (IBM Corp., Armonk, NY, USA). Two-sided tests of significance *p* < 0.05 were used in all analyses.

## 3. Results

### 3.1. Patient Characteristics

To find miRNAs that characterize SSc-PAH, we extracted RNA from SSc patients with and without PAH. The detailed clinical features of these patients are presented in Table 1. There was no significant difference in the duration of illness between the groups. However, SSc-PAH patients were diagnosed with SSc at an older age than SSc-noPAH patients (65 years vs. 46 years, *p* < 0.05). Furthermore, as expected, median echo pulmonary artery pressure results (mPAP) were higher in the SSc-PAH than in the SSc-noPAH group (65 mmHg vs. 28 mmHg, respectively, *p* < 0.05; Table 1). Median diffusing capacity of lung for carbon monoxide (DLCO) and six-minute walk (6 MW) test results were lower in the SSc-PAH group (DLCO: 43% vs. 59% and 6 MW: 259 m vs. 513 m (*p* < 0.05 for both), further detailed in Table 1). Among the laboratory tests, only the median level of anti-centromere antibodies differed between groups, with higher levels in the SSc-PAH than in the SSc-noPAH group (positive in 83% vs. 12%, respectively, *p* < 0.05).

### 3.2. Literature Search: Circulating miRNAs That May Differentiate between SSc and SSc-PAH Patients

Previous studies have demonstrated that miRNAs are involved in the development of SSc. Therefore, we examined the literature and generated a list of seven miRNAs. The seven miRNAs were chosen following a literature search for miRNAs that were (a) found in the circulation of SSc-PAH or SSc-noPAH patients (b) associated with fibrosis (c) correlated with disease prognosis. Table 2 indicates the miRNAs, biological material (circulating blood/cells) and what was reported in these studies. We hypothesized that some of these miRNAs may distinguish between SSc patients with and without PAH.

### 3.3. miRNA Pattern in SSc-noPAH Versus SSc-PAH Patients

**Figure 1:**
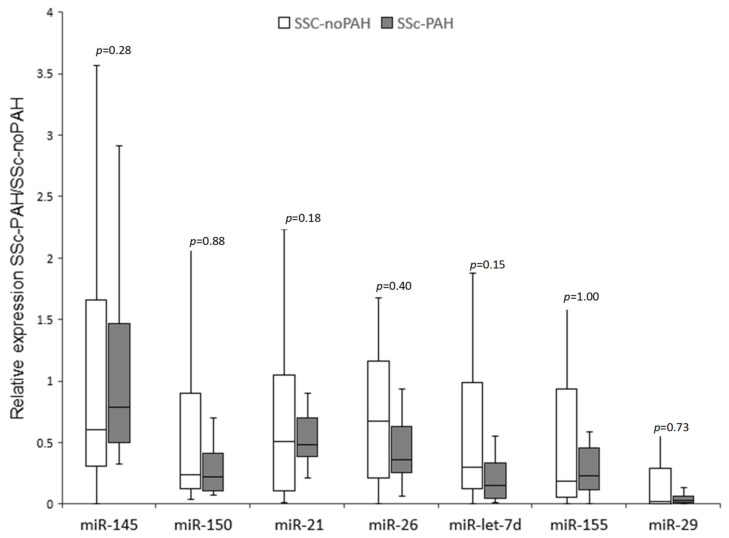
Median miRNA levels in the plasma of SSc patients with and without PAH. RNA was isolated from plasma of 25 SSc-noPAH and 12 SSc-PAH patients. The levels of seven miRNAs were evaluated by qRT-PCR. miR-16 served for normalization. The boxplot presents five values (minimum: (Q1–1.5*IQR); first quartile (Q1/25th percentile); median; third quartile (Q3/75th percentile); and maximum (Q3 + 1.5*IQR)) of the miRNAs. miR: miRNA; PAH: Pulmonary arterial hypertension; SSc-noPAH: Patients with SSc without PAH; SSc-PAH: Patients with SSc and PAH.

**Figure 2:**
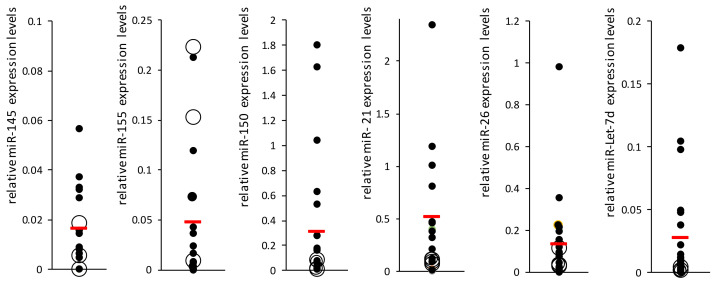
Mean miRNA levels in the SSc-noPAH group. miRNA levels in the plasma of 25 SSc-noPAH patients were evaluated using qRT-PCR. miR-16 served for normalization. Patients underwent echocardiography before blood sampling and at the end of the study. miRNA values of the patients with stable echo results during this time are marked in full black dots (*n* = 21). The miRNA levels of patients whose echo levels increased during this period are marked by empty circles (*n* = 4). All y-axis values are presented relative to miR-16. The red line indicates the mean value of each group.

To evaluate the levels of the miRNAs shown in Table 2 in the circulating blood of SSc-noPAH and SSc-PAH patients, we isolated RNA from patients’ plasma. First, we measured the level of the control miR-16 in both groups using qRT-PCR. As previously described [11,12], we found similar levels in both groups (SSc-noPAH CT = 25.8, SSc-PAH CT = 26.1) and concluded that miR-16 can serve as a normalizing miRNA. Then, we measured the levels of the seven miRNAs relative to the normalizing miR-16 using qRT-PCR (Figure 1). miR-23 was not detected in the patients’ plasma. miR-29 levels were very low in the plasma and PCR results were found in only about 60% of the samples; therefore, it was excluded from the analysis. The most significant difference in miRNA expression between the two groups was that of miR-let-7d, which was decreased in the PAH group (*p* = 0.15, Figure 1). miR-let-7d levels were homogeneous in the PAH group. Nevertheless, high heterogeneity was found between the levels of all other miRNAs, both in the SSc-PAH group and especially in the SSc-noPAH group. Since the miRNA levels varied greatly, particularly in the SSc-noPAH group, as described in Figure 2, we suspected that the physiological conditions of the patients in this group were not consistent and that among the SSc-noPAH patients who were not diagnosed with SSC-PAH, were some with early onset PAH. Therefore, we assumed that this subset of patients expressed miRNAs at different levels than those who had not yet developed symptoms of PAH.

SSc patients undergo routine monitoring to evaluate their condition every few months. One of the tests used is echocardiography (echo), which measures mean pulmonary artery pressure; elevated levels imply disease development. Therefore, data from patients in the SSc-noPAH group (from the time of blood sample collection for miRNA analysis until the end of the experiment, ~1–2 years) were analyzed to assess whether changes in their echocardiograms might indicate the development of PAH. Among the SSc-noPAH patients, we found three whose mean pulmonary arterial pressure had increased during the study by >5 mmHg and one whose pulmonary pressure could not be assessed using echo due to technical issues (lack of tricuspid regurgitation) but had developed PAH. miRNA levels of all patients are shown in Figure 2 as full black dots and the four patients with increased pulmonary artery pressure or who developed PAH (described above) are marked as an empty larger circle. Interestingly, it was found that the levels of four miRNAs (miR-let-7d, miR-26, miR-150 and miR-21) in these four patients (empty circles) were relatively low and similar to those of PAH patients. Among these four patients, two developed PAH.

As the four patients whose medical condition deteriorated during the study had similar miRNA levels as the SSc-PAH patients, but were similar in other parameters (such as lung function) to those of SSc patients who did not have PAH, we termed them SSc-susPAH (suspected PAH). We decided to exclude this subset of patients from both groups and from further analysis.

Additional statistical tests (Mann–Whitney) that compared the miRNA levels (21, 7, 150, 155, 145 and 26) of the SSc-noPAH group (without SSc-susPAH) and the PAH group, demonstrated a significant decrease in the level of miR-let-7d (*p* < 0.05) and a trend toward decreased levels of miR-26 (*p* = 0.14) in the PAH group.

Moreover, Spearman correlation tests were used to examine whether there was a relation between development of PAH and the patients’ demographic parameters, the presence or absence of autoantibodies (anti-centromere antibody, anti-DNA-topo-isomerase I and anti-RNA-polymerase III), CRP levels, the level of miRNAs in the plasma and the pulmonary function test results (DLCO, 6MW). We found positive correlations between PAH and age (rs = 0.56, *p* < 0.05), anti-centromere antibody positivity (rs = 0.67, *p* < 0.05) and, as expected, to pulmonary artery pressure (mPAP, rs = 0.82, *p* < 0.05). A negative correlation was found with miR-let-7d plasma levels (rs = -0.41, *p* < 0.05) and as expected, with lung function tests (DLCO: rs = -0.53, *p* < 0.05, 6MW: rs = -0.55, *p* < 0.05).

We also performed a partial Spearman’s Rho correlation between the PAH complication and the miRNAs, placing age and sex as confounding factors. We chose only these confounders as limiting factors because other parameters that were correlated with PAH (previous section, lung function and mPAP) are part of the definition of the disease. Partial Spearman’s Rho correlation, which measures the association between the PAH phenotype to miR-let-7d and miR-26 levels, while adjusting for age, showed that these correlations were significant and not age-dependent (*p* = 0.011 and *p* = 0.045, respectively).

### 3.4. Partial Spearman’s Rho Correlation between miRNA Level and Laboratory Parameters

We hypothesized that antibodies and complement might contribute to the generation of the pro-inflammatory/profibrotic tissue of patients. We also assumed that these changes would be early events in the disease and might be affected by the miRNAs. Therefore, we examined whether there were correlations between miR-let-7d and miR-26 and the patients’ laboratory parameters (C3, C4, CRP, IgG1, IgG2, IgG3, IgG4, IgG and autoantibodies (anti-centromere, anti-DNA-topo-isomerase I and anti-RNA-polymerase III)). A partial Spearman’s Rho correlation that measured the relations between levels of miR-let-7d and miR-26, between miR-let-7d and IgG4, between miR-let-7d and CRP and between miR-26 and IgG2, while adjusting for age and sex, showed that all correlations were statistically significant (*p* < 0.05) and were not conditional on age or sex (Table 3). Furthermore, a significant partial correlation was found between miR-26 level and C3 (*p* < 0.05) and a trend between miR-let-7d and C3 (*p* = 0.07), while adjusting for age, sex and C4 (which is correlated with C3, as described in Table 3).

Positive correlations were found between miR-26 and miR-let-7d and the C3 level. Since the miRNA levels were lower in PAH patients, we examined whether the level of C3 in the blood of SSc-PAH and SSc-susPAH patients was also lower than in SSc-noPAH patients. We found that the mean C3 level was 109 mg/dL among the SSc-noPAH patients, 97 mg/dL among the SSc-PAH patients and 87 mg/dL in the SSc-susPAH group. While the directionality of these results is as expected, the differences did not reach statistical significance, perhaps, due to the small cohort size.

In conclusion, these data suggest that there is an association between low expression of miR-26 and miR-let-7d and the PAH complication, and that there is a correlation between these miRNAs, and parameters associated with inflammation (CRP) and complement activation. These associations may suggest that these miRNAs are involved in mediating the activity of the complement system. These assumptions will need to be proven in future studies, using appropriate biological systems.

### 3.5. Finding Targets of miR-Let-7d and miR-26 Involved in Immune, Endothelial and Myofibroblast Cell Biology

We surmised that if we identify known targets of let-7 and miR-26 that are also relevant to the cell biology of the major players in SSc, we may learn how these miRNAs contribute to its development. Therefore, we conducted a bioinformatics analysis of the microRNAs’ targets using the software packages targetscan (http://www.targetscan.org, accessed on 30 October 2021) and mirbd (http://mirdb.org, accessed on 30 October 2021).

The resulting lists for each miRNA were combined to include all implicated genes (461 for miR-let-7d and 1,112 for miR-26). We also collected established characteristic gene lists for immune cells (233 genes), endothelial cells (130 genes) and myofibroblasts (477 genes). Using Venny software, we found genes common to each cell type with each miRNA (yielding six lists of 7–30 genes, Table 4 and Table 5). Then, we used Toppgene software to find GO biological annotations that match each of the six lists. We speculated that the annotations would suggest biological processes in which the miRNAs are involved. The results shown in Table 4, Table 5, Tables S1 and S2 suggest that both miRNAs are involved in cytokine signaling (miR-let-7d in IL-10 and CCR7 and miR-26 in IL18R1 and IRF4). Moreover, the analysis suggests that miR-26 is involved in blood vessel biology and in motility processes, while miR-let-7d appears to be significant in the processes of myofibroblast proliferation and endothelial cell proliferation and apoptosis (Tables S1 and S2). Our bioinformatics study also showed that both miRNAs regulate genes that belong to the TGF-β signaling pathway or to the SMAD binding gene sets (M39432 and GO:0046332, respectively; genes are highlighted in gray in Table 4 and Table 5) but mostly in relation to the myofibroblast cells. Moreover, miR-let-7d regulates the expression of endothelin 1 gene (EDN1, bold in Table 4). Endothelin 1 (ET-1) is a potent vasoconstrictor that is elevated in the plasma of patients with PAH [26], is known to potentiate the induced effect of TGF-β on mesenchymal cells and on the expression of profibrotic genes and proteins.

The complement pathway did not appear in the annotations because the gene lists of the immune system cells contained C3, while the gene lists of miR-26 and miR-let-7d contained the complement components C1 and C2.

### 3.6. WBCs of SSc-noPAH and SSc-PAH Patients Have Equal Levels of miR-26 and miR-let-7d

**Figure 3:**
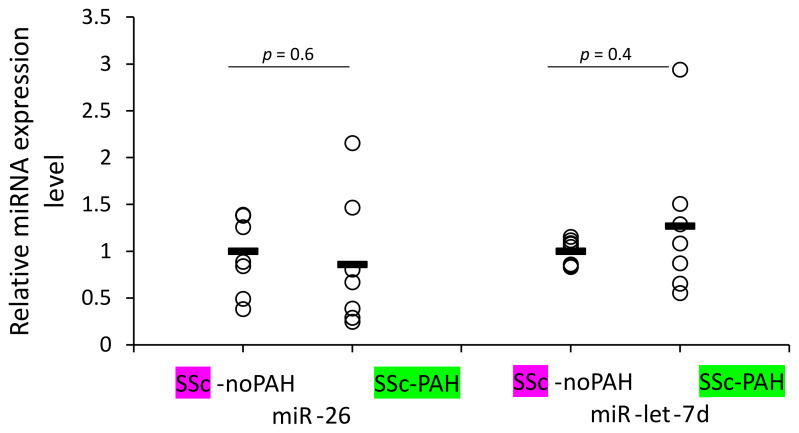
miR-26 and miR-let-7d levels in WBCs of SSc-noPAH and SSc-PAH patients. WBCs were isolated from 7 SSc-PAH and 8 SSc-noPAH patients. RNA was isolated from the cells and the levels of miR-26 and miR-let-7d were evaluated with qRT-PCR. miR-16 served as the normalizing miRNA. The graph represents miRNA expression levels relative to miR-16. Horizontal bars represent the mean values of the miRNAs.

We aimed to identify which cells are involved in mediating the reduced levels of miR-26 and miR-let-7d expression found in the plasma of SSc-PAH patients. Our clinical correlations suggested involvement of miRNAs in activating the complement system and antibody levels (levels of both miRNAs correlated with C3 and miR-26 also with IgG2 levels) and the bioinformatic analysis suggested involvement of the miRNAs in cytokine signaling. These factors are mediators of the immune system. Therefore, we primarily opted to find whether WBCs of SSc-PAH patients contain lower miR-26 and miR-let-7d levels compared to those of SSc-noPAH patients who do not have PAH. Unexpectedly, we found that they express equal amounts of the miRNAs (Figure 3).

### 3.7. Exposure of Dermal Fibroblasts to Tgf-β and Their Differentiation into Myofibroblasts Was Accompanied by Decreased miR-Let-7d and miR-26 Levels

Myofibroblasts induce a severe fibrotic process, affecting the fibroproliferative changes in the skin, internal organs and pulmonary arterioles in SSc [27,28]. They originate from resident fibroblasts, bone marrow-derived cells and the conversion of endothelial cells into activated fibroblasts [28]. A key growth factor for myofibroblast formation is TGF-β, which induces collagen production and alpha smooth muscle actin (αSMA) expression in the fibroblasts [3]. The bioinformatic analysis suggested that miR-let-7d and miR-26 regulate genes involved in the TGF-β pathway, especially in myofibroblast cells (Table 4 and Table 5). 

**Figure 4:**
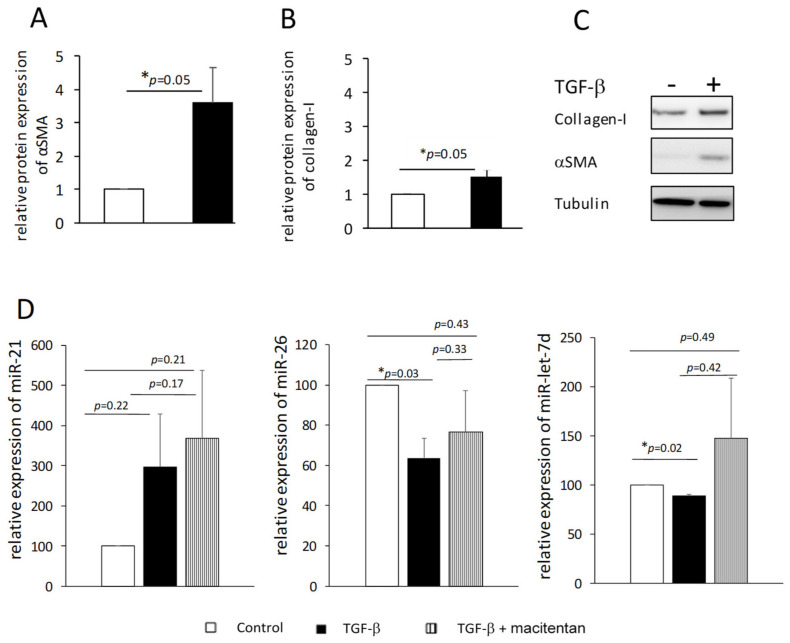
TGF-β induced myofibroblast differentiation is accompanied by reduced miR-26 and miR-let-7d levels. Dermal fibroblasts were isolated from healthy patients undergoing plastic surgery. The cells were exposed to TGF-β (10 ng/mL) for 48 h or 72 h. Then the cells were either harvested and counted (72 h) or harvested and their proteins and RNA isolated (48 h). The levels of the myofibroblast markers αSMA (**A**) and collagen I (**B**) were assessed by western blot, and the levels of miR-21, miR-26 and miR-let-7d were assessed with qRT-PCR (**D**). (**C**) is a representative blot of αSMA and collagen in cells that were treated or not treated with TGF-β (*n* = 4). Columns represent mean and whiskers represent standard errors. *—Statistically significant results, *p* ≤ 0.05.

We suspected that exposing fibroblasts to TGF-β might reduce the levels of miR-26 and miR-let-7d in the cells and their secretion in the circulation. Therefore, we isolated dermal fibroblasts from normal skin biopsies, exposed them to TGF-β and induced their differentiation into myofibroblasts, as was evident by the increase in the number of cells (40%↑, 72 h, *p* < 0.05, data not shown) and increased expression of αSMA and collagen I (48 h, *p* = 0.05, Figure 4A–C). Total RNA was isolated from the cells and miR-26 and miR-let-7d levels were measured. As an additional control, we measured the level of miR-21, which is a pro-fibrotic miRNA [29]. We found small but consistent decreases in miR-let-7d levels (~10%, *p* < 0.05) and ~40% reduction in the level of miR-26 (*p* < 0.05) compared to cells that were not exposed to TGF-β, whereas miR-21 levels tended to increase in those cells (Figure 4D, black and white bars, respectively). These results support our hypothesis that the presence of low levels of miR-26 and miR-let-7d in the bloodstream is at least partly due to their low production by myofibroblast cells.

### 3.8. The Effect of Macitentan (ET-1 Receptor Antagonist) on Myofibroblast miR-26 and miR-Let-7d Levels

Dermal fibroblasts produce endothelin-1 (ET-1) [27], which plays a key role in the biology of PAH [30]. Previous publications showed that macitentan, an ET-1 receptor antagonist approved for PAH therapy, interferes with ET-1 and TGF-β-induced fibroblast activation [27]. We examined whether exposing fibroblasts cultured with TGF-β to macitentan affects the levels of miR-26 and miR-let-7d, and found that the addition of the drug together with TGF-β prevented the decrease in the levels of miR-26 and miR-let-7d observed when the cells were exposed to TGF-β only (Figure 4D).

## 4. Discussion

The idea of using miRNAs as biological markers has been suggested in the past in the context of various pathologies [31]. In the current study, we searched for miRNAs that are expressed differently in SSc-PAH compared to SSc-noPAH patients. Our initial observation was that miRNA expression levels vary greatly among patients with SSc-noPAH. A more thorough examination of the SSc-noPAH group showed that while most SSc-noPAH patients presented with a stable mean pulmonary artery pressure, some had a relatively rapid rise in this variable between their first and last echo tests included in this study. These patients were termed SSc-susPAH (suspected to develop PAH). A comparison between SSc-noPAH and SSc-PAH patients showed differences in age at diagnosis, clinical parameters related to the disease (echo and lung function tests), anti-centromere levels and in the levels of miR-let-7d and miR-26. These clinical differences were similar to those described in the literature [4,32]. To the best of our knowledge, this is the first study to show that miR-26 levels in the plasma of SSc-PAH patients is lower than in the plasma of SSc-noPAH patients. Regarding miR-let-7d, our observations agree with those of a study by Wuttge et al. that showed that the level of miR-let-7d is lower in SSc-PAH compared to SSc-noPAH patients [33].

Echocardiography, which is a recommended modality for early detection of PAH, was used to differentiate between SSc-noPAH and SSc-susPAH patients [34]. Even a borderline elevation in mean pulmonary artery pressure from 21 to 24 mmHg may reflect an intermediate stage on the continuum between normal pressure and PAH [35]. Previous publications also showed that SSc patients with increasing systolic pulmonary artery pressure over a short period have a higher risk of mortality from PAH [7,36]. We found three SSc-susPAH patients with increased mean pulmonary artery pressures in the one- to two-year period between the initial blood collection and the end of the study. Interestingly, in these patients and in another SSc-noPAH patient whose mean pulmonary artery pressure at the end of the study could not be evaluated by echo due to technical problems, plasma levels of miR-let-7d and miR-26 were similar to those measured in SSc-PAH patients and were significantly lower than those measured in SSc-noPAH patients. At the end of the study period, two of these four patients developed PAH and one died from this complication. These results suggest that low levels of miR-let-7d and miR-26 miRNAs that characterize SSc-PAH patients, can be detected in SSc-noPAH patients even before pulmonary pressure rises. Despite this interesting observation, the limited number of patients (4) does not allow unequivocal conclusions to be drawn. A future, multicenter study with a larger patient population may reconfirm these findings.

To further assess the involvement of miR-let-7d and miR-26 in the pathogenesis of SSc and PAH, miRNA levels and the clinical and laboratory data of the patients were correlated. Interestingly, we found that the miR-let-7d and miR-26 levels correlated with C3. Using the http://mirdb.org database, we found that C2 is a target of miR-let-7d and C1s is a target of miR-26. Protease C1s cleaves C4 and C2 to generate the C3 convertase that cleaves C3, resulting in a decrease in C3. These processes may explain the positive correlation between miR-let-7d and miR-26 levels and C3. The relation between activation of the complement system to PAH was only recently suggested by demonstrating that vascular-specific, immunoglobulin-driven dysregulated complement signaling triggers and maintains pulmonary vascular remodeling and PAH [37]. It was also suggested that plasma complement signaling, including factors in the alternative pathway, is a prognostic factor for survival in patients with idiopathic PAH. 

As a correlation was found between the miR-let-7d and miR-26 and C3 and between miR-26 and IgG2 that can activate the classic complement system, it was tempting to speculate that the lower levels of miR-26 and miR-let-7d are an early event in the development of SSc-PAH, at the stage of autoimmune attack. Our bioinformatic search also suggested involvement of these miRNAs in mediating the immune response in SSc. Chemokine (C-C Motif) Receptor 7 (CCR7), a miR-let-7d target expressed by WBCs, was previously found to mediate PAH in murine models. C-C Motif Chemokine Ligand 21 (CCL21) the ligand of CCR7, is a promising marker for predicting the risk of SSc-related PAH and PAH progression [38]. Furthermore, interleukin-18 (IL-18) Receptor 1 (IL-18R1) is a miR-26 target expressed by WBCs. Previous publications showed that IL-18 levels are altered in SSc patients [39,40] and that there is a positive correlation between serum IL-18 binding protein (IL-18BPa) levels and right ventricular systolic pressure [39].

The clinical correlations and bioinformatic analysis encouraged us to check whether miR-let-7d and miR-26 levels in WBCs of SSc-noPAH patients are higher than in SSc-PAH patients. Contrary to our expectations, similar levels of the miRNAs were found in the cells. This suggests that the low level of miRNAs in the circulating blood of PAH patients is not due to decreased production by WBCs. We performed the test on a mixed population of WBCs that was not separated into the different types of immune system cells. miR-26 or miR-let-7d levels might be reduced in one of the immune cell populations and the low levels might be masked by their expression in other WBCs; a possibility that should be explored in the future.

Our bioinformatic analyses also suggested that miR-26 might be involved in blood vessel morphogenesis and cell motility. While we did not continue our study in this direction, it is tempting to think that this miRNA is involved in the process of endothelial mesenchymal transition (EndMT). This process demands acquisition of mesenchymal characteristics by endothelial cells, such as cell migration [41] and ends with altered vessel morphology. It has been reported that EndMT may also have a pivotal role during PAH pathogenesis, promoting endothelial cell dysfunction [42]. Our bioinformatic analysis also suggested that miR-let-7d and miR-26 are involved in the biology of myofibroblast cells by regulating genes in the TGF-β and endothelin pathways. This is in accordance with literature that showed that collagen, a target protein of both miRNAs, is regulated by the TGF-β pathway [29]. This suggests that exposure of fibroblasts to TGF-β and/or endothelin 1, might alter miR-let-7d and miR-26 production. Indeed, we found that TGF-β significantly reduced the level of both miRNAs in dermal fibroblasts. These observations suggest that myofibroblasts are at least partially responsible for reduced miR-let-7d and miR-26 levels in the plasma of SSc-PAH patients. This agrees with reports that showed that miR-let-7d and miR-26 expression are downregulated in the skin of SSc patients [29,43] and that miR-let-7d is reduced in the skin of SSc-PAH patients even more than it is among SSc-noPAH patients [23]. 

Additionally, we found a correlation between the two microRNAs (miR-let-7d and miR-26). An interaction between these two miRNAs was previously described [44].in publication who showed that miR-26 disrupts the Lin28B/miR-let-7d circuit [44]. The Lin28B gene is negatively regulated by miRNAs (such as miR-26) and its overexpression is linked to the repression of let-7 miRNAs [44]. A previous publication also showed that Lin28b serves as a molecular switch for tuning the tolerance threshold during B cell receptor (BCR) repertoire selection early in life [45]. Therefore, it is enticing to speculate that this protein may contribute to the development of SSc, in which the appearance of autoantibodies is the first clinical phenomenon observed. 

This study examined the level of miRNAs in patients treated in the rheumatology clinic at our medical center and its major limitation is the small cohort. An additional limitation is that only seven miRNAs were tested, while previous articles showed involvement of additional miRNAs in PAH and systemic sclerosis.

Despite the small group of patients, our results suggest that miR-let-7d and miR-26 are serological markers that differentiate SSc-noPAH from SSc-PAH patients. Moreover, decreased expression of the miRNAs among SSc-susPAH patients also suggests that they may be early predictors of the development of PAH, an assumption that warrants further testing with larger cohorts.

## Figures and Tables

**Table 1:** Demographic and clinical characteristics of the patients.

Demographic Parameters	SSc-noPAH (*n* = 25)		SSc-PAH (*n* = 12)	
	Valid N	Median (25–75 Percentile)/%	Valid N	Median (25–75 Percentile)/%
Female (a)	25	88%	12	83%
Age at diagnosis (years) (b)	25	46.0 (39.0–57.0) *	12	65.0 (61.0–73.0)
Duration of illness (years) (b)	25	6.0 (4.0–12.0)	12	3.5 (2.0–9.0)
Clinical parameters				
Diffuse vs. limited SSc (a)	25	92% *	12	42%
Echo (mPAP, mmHg) (b)	25	28.0 (25.0–30.0) *	12	65.0 (49.5–82.0)
FVC (%) (b)	18	82.0 (72.0–101.0)	10	70.5 (58.0–84.0)
FEV1 (%) (b)	18	82.5 (71.0–95.8)	10	64.0 (53.0–75.0)
DLCO (%) (b)	18	59.3 (51.3–68.0) *	9	43.0 (31.0–48.3)
6 MW (meter) (b)	17	513.0 (456.0–528.0) *	8	259.5 (151.0–430.0)
C-reactive protein CRP mg/dl (b)	22	0.8 (0.3–1.5)	12	0.8 (0.4–1.8)
Anti-centromere (a)	8	12% *	6	83%
Anti-DNA-topo-isomerase I (a)	24	S137%	12	17%
Anti-RNA-polymerase III (a)	24	33%	11	27%

Median and 25–75 percentile/% were used to demonstrate the distribution of non-categorical variables and % for the categorical variables. (a) Chi-square test was used to test relationships between categorical variables and the two independent groups. (b) Mann–Whitney U test was used to compare differences between the two independent groups: SSc-noPAH and SSc-PAH. DLCO: Diffusing capacity of lung for carbon monoxide; Echo: Echocardiogram; FEV1: Forced expiratory volume in one second; FVC: Forced vital capacity; 6 MW: Six-minute walk; * Results significantly different from SSc-PAH, *p* < 0.05.

**Table 2:** miRNAs that characterize pulmonary fibrosis, PAH or are involved in the development of SSc.

miRNA	Previous Knowledge	Biological Material	References
miR-23a	Biomarkers in idiopathic PAH	Circulating blood	[13]
miR-29a	Increased in SSc Induce ventricular hypertrophy and fibrosis	Circulating blood Skin	[14,15,16]
miR-26a	Reduced in patients with PAH	Circulating blood	[17]
miR-150	Reduced level is associated with poor survival in PAH	Circulating blood Dermal fibroblasts	[18,19]
miR-21	Enhances vascular cell proliferation	Circulating blood	[20,21,22]
miR-let-7d	Negatively correlated with severity of PAH in patients with SSc	Skin Circulating blood	[23,24]
miR-155	Drives fibrosis	Fibroblasts	[25]

miR: miRNA; PAH: Pulmonary arterial hypertension; SSc: Systemic sclerosis.

**Table 3:** Correlation tests between miRNA levels and IgG and C3/4 levels.

Variables		Confounders-Z	r(s)xy.z	*p*-Value	*n*
X	Y				
miR-let-7d	miR-26	Age, Sex	0.431	0.006	31
miR-let-7d	C3	Age, Sex, C4	0.437	0.070	16
miR-let-7d	IgG4	Age, Sex	−0.626	0.017	12
miR-let-7d	CRP	Age, Sex	0.342	0.032	28
miR-26	C3	Age, Sex, C4	0.468	0.050	16
miR-26	IgG2	Age, Sex	−0.626	0.017	12

C3: Complement component 3; C4: Complement component 4; CRP: C-reactive protein; miR: miRNA; *n*: Number of patients; r(s)xy.z = partial Spearman’s Rho correlation coefficient.

**Table 4:** Genes controlled by miR-let-7d and involved in the biology of immune, myofibroblast and endothelial cells.

	miR-let-7d (461 Genes)	
Cells (Number of Genes)	Number of Genes	Genes in Venny
**Immune cells (233 genes)**	16	*CCR7*, *FAS*, *EDN1*, *RAG1*, *ICOS*, *FASLG*, *IL10*, *RORC*, *MAPK8*, *MASP1*, *NAP1L1*, *TAB2*, *BCL2L1*, *COL4A5*, *IL13*, *IL6*
**Myofibroblasts (447 genes)**	19	*COL1A2*, *RANBP2*, *TGFBR1*, *SCN5A*, *IGF1*, *TP53*, *CASP3*, *ITGB3*, *COL3A1*, *NGF*, *HAS2*, *P4HA2*, *MAP8*, *CCND1*, *CDKN1A*, *SMUG1*, *CCL7*, *IL10*, *HMGA2*, *EDN1*
**Endothelial cells (130 genes)**	7	*ITGP3*, *TNFRSF1*, *CASP3*, *FASLG*, *MAPK8*, *EDN1*, *PIK3CA*, *CCND2*, *COL4A1*, *COL1A2*, *COL3A1*

Venny software was used to find genes common to miR-let-7d targets and to the lists of the three types of cells (immune, myofibroblast and endothelial cells) yielding three lists of genes. Genes marked in gray belong to the “TGF-β signaling pathway” or to the “SMAD binding” gene sets.

**Table 5:** Genes controlled by miR-26 and involved in the biology of immune, myofibroblast and endothelial cells.

	miR-26 (1112 Genes)	
**Cells (Number of Genes)**	Number of Genes	Genes in Venny
**Immune cells (233 genes)**	8	*MAP3K7*, *TRAF3*, *IRF4*, *SELP*, *TAB3*, *ILI8R1*, *ICOS*, *PYGS2*
**Myofibroblasts (447 genes)**	30	*HAS2*, *GSK3B*, *COL1A2*, *PCNA*, *SCN5A*, *CREB1*, *LTBP1*, *COL12A1*, *PTEN*, *JAG1*, *ESR1*, *PALLD*, *KCNJ2*, *PAK2*, *MLANA*, *SNTG1*, *SCL25A16*, *HMGA2*, *HDAC9*, *MIB1*, *PGR*, *MYLK3*, *SFTPB*, *ROCK1*, *WNT5A*, *MAP3K2*, *SMAD4*, *PURA*, *PURB*, *TMTC3*
**Endothelial cells (130 genes)**	7	*ADAM17*, *SELP*, *CDH2*, *PTGS2*, *PECAM1*, *EREG*, *PDGRFRA*

Venny software was used to find genes common to miR-26 targets and to the lists of the three types of cells (immune, myofibroblast and endothelial cells), yielding three lists of genes. Genes marked in gray belong to the “TGF-β signaling pathway” or to the “SMAD binding” gene sets.

## Data Availability

Data are contained within the article except for the bioinformatics analysis data extracted from the computer sites described in the Methods and Materials (Bioinformatics) sections.

## Supplementary Materials

The following supporting information can be downloaded at: https://www.mdpi.com/article/10.3390/biomedicines10030629/s1, Table S1: GO biological annotations for genes common to the miR-let-7d targets and to the lists of genes of immune, endothelial and myofibroblast cells; Table S2: GO biological annotations for genes common to the miR-26 targets and to the lists of genes of immune, endothelial and myofibroblast cells.

## Abbreviations

αSMA	alpha smooth muscle actin
ACR	American College of Radiologists
BCR	B cell receptor
BP	Binding protein
CCL21	C-C Motif Chemokine Ligand 21
CCR7	C-C Motif chemokine Receptor 7
CT	Cycle threshold
DLCO	Diffusing capacity of lung for carbon monoxide
DMEM	Dulbecco’s Modified Eagle Medium
DMSO	Dimethyl sulfoxide
DTT	Dithiothreitol
Echo	Echocardiography
EDN1	Gene symbol of endothelin 1
EDTA	Ethylenediaminetetraacetic acid
EndMT	Endothelial mesenchymal transition
ET-1	Endothelin 1
EULAR	European League Against Rheumatism
Fev1	Forced expiratory volume in one second
Fvc	Forced vital capacity
GO	Gene ontology
h	Hour
hsa	Homo sapiens
IgG	Immunoglobulin G
IL	Interleukin
IRF4	Interferon Regulatory Factor 4
miRNA, miR	microRNA
PAH	Pulmonary arterial hypertension
PBS	Phosphate Buffered Saline
PVDF	membrane: Poly(vinylidene fluoride)
SSc	Systemic sclerosis
SSc-noPAH	SSc patients without PAH
SSc-PAH	SSc patients with PAH
SSc-susPAH	SSc patients suspected to develop PAH
qRT-PCR	Real time quantitative polymerase chain reaction
rs	Spearman’s Rank calculation
RT cDNA	Reverse transcription complementary DNA
SDS	Sodium dodecyl sulfate
TGF-β	Transforming growth factor beta
Tris	Tris(Hydroxymethyl)aminomethane
WBCs	White blood cells
