# Supplementary material for: MiRNAs in Systemic Sclerosis Patients with Pulmonary Arterial Hypertension: Markers and Effectors
Source: Biomedicines. 2022 Mar 8;10(3):629. doi: 10.3390/biomedicines10030629 (PMC8945806; doi:10.3390/biomedicines10030629)
Supplement: Supplementary file 1 [file biomedicines-10-00629-s001.zip › biomedicines-1540269-supplementary.pdf]

biomedicines

Table S1. GO biological annotations for genes common to the miR-let-7d targets and to the lists of genes of immune, endothelial and myofibroblast cells.

miR-let-7d	ID	Name	Bonferroni	Genes from Input	Genes in Annotation
**Immune system**					
1	GO:0032496	response to lipopolysaccharide	3.81×10^{-7}	6	368
2	GO:0002237	response to molecule of bacterial origin	5.32×10^{-7}	6	389
3	GO:0033993	response to lipid	9.54×10^{-7}	7	977
4	GO:0071345	cellular response to cytokine stimulus	3.94×10^{-6}	7	1196
5	GO:0034097	response to cytokine	6.71×10^{-6}	7	1290
6	GO:0051090	regulation of DNA-binding transcription factor activity	3.52×10^{-5}	6	782
7	GO:0019221	cytokine-mediated signaling pathway	4.74×10^{-5}	6	822
8	GO:0009617	response to bacterium	5.06×10^{-5}	6	831
9	GO:1901700	response to oxygen-containing compound	9.24×10^{-5}	7	1875
10	GO:0071887	leukocyte apoptotic process	1.01×10^{-4}	4	134
**Myofibroblasts**					
1	GO:0072359	circulatory system development	6.01×10^{-8}	13	1340
2	GO:0009719	response to endogenous stimulus	9.97×10^{-8}	14	1780
3	GO:0050679	positive regulation of epithelial cell proliferation	1.23×10^{-7}	8	245
4	GO:0008284	positive regulation of cell population proliferation	1.70×10^{-7}	12	1117
5	GO:0042127	regulation of cell population proliferation	3.99×10^{-7}	14	1973
6	GO:0010165	response to X-ray	4.68×10^{-7}	5	35
7	GO:0071363	cellular response to growth factor stimulus	2.25×10^{-6}	10	775
8	GO:0070848	response to growth factor	3.25×10^{-6}	10	805
9	GO:0034349	glial cell apoptotic process	4.38×10^{-6}	4	17
10	GO:0051338	regulation of transferase activity	4.50×10^{-6}	11	1131
**Endothelial cells**					
1	GO:0060548	negative regulation of cell death	4.44×10^{-6}	7	1214
2	GO:0070848	response to growth factor	4.24×10^{-5}	6	805
3	GO:0051247	positive regulation of protein metabolic process	4.78×10^{-5}	7	1704
4	GO:0032496	response to lipopolysaccharide	6.35×10^{-5}	5	368
5	GO:0002237	response to molecule of bacterial origin	8.37×10^{-5}	5	389
6	GO:1901214	regulation of neuron death	1.04×10^{-4}	5	406
7	GO:0010941	regulation of cell death	1.24×10^{-4}	7	1952
8	GO:0042127	regulation of cell population proliferation	1.34×10^{-4}	7	1973
9	GO:0070997	neuron death	1.69×10^{-4}	5	448
10	GO:0071345	cellular response to cytokine stimulus	4.51×10^{-4}	6	1196

GO biological annotations of the 3 groups of genes found in Table 4. The annotations were divided into different categories that were marked by different colors. Explanations for the colors: Gray-response to bacteria, Yellow-response to cytokine, Blue-cell proliferation and response to growth factor, Violet-circulatory system and tube biology, Red-cell death.

Table S2. GO biological annotations for genes common to the miR-26 targets and to the lists of genes of immune, endothelial and myofibroblast cells.

miR-26	ID	Name	Bonferroni	Genes from Input	Genes in Annotation
**Immune system**					
1	GO:0071345	cellular response to cytokine stimulus	1.48×10^{-5}	7	1196
2	GO:0034097	response to cytokine	2.50×10^{-5}	7	1290
3	GO:0019221	cytokine-mediated signaling pathway	9.4×10^{-5}	6	822
4	GO:0002682	regulation of immune system process	2.05×10^{-4}	7	1747
5	GO:0002224	toll-like receptor signaling pathway	2.33×10^{-4}	4	166
6	GO:0006952	defense response	4.17×10^{-4}	7	1935
7	GO:0010647	positive regulation of cell communication	4.43×10^{-4}	7	1952
8	GO:0023056	positive regulation of signaling	4.49×10^{-4}	7	1956
9	GO:1902533	positive regulation of intracellular signal transduction	5.93×10^{-4}	6	1129
10	GO:0002221	pattern recognition receptor signaling pathway	7.33×10^{-4}	4	221
**Myofibroblasts**					
1	GO:0007507	heart development	4.02×10^{-10}	14	698
2	GO:0072359	circulatory system development	1.06×10^{-8}	16	1340
3	GO:0009887	animal organ morphogenesis	1.14×10^{-6}	14	1263
4	GO:0001837	epithelial to mesenchymal transition	9.36×10^{-6}	7	169
5	GO:0060429	epithelium development	1.42×10^{-5}	14	1532
6	GO:0035295	tube development	2.38×10^{-5}	13	1310
7	GO:0035239	tube morphogenesis	2.78×10^{-5}	12	1068
8	GO:0009719	response to endogenous stimulus	9.70×10^{-5}	14	1780
9	GO:0071495	cellular response to endogenous stimulus	1.30×10^{-4}	13	1510
10	GO:0048762	mesenchymal cell differentiation	1.65×10^{-4}	7	256
**Endothelial cells**					
1	GO:2000145	regulation of cell motility	2.64×10^{-4}	6	1159
2	GO:0040012	regulation of locomotion	3.41×10^{-4}	6	1210
3	GO:0051270	regulation of cellular component movement	4.14×10^{-4}	6	1250
4	GO:0042060	wound healing	4.87×10^{-4}	5	594
5	GO:0050731	positive regulation of peptidyl-tyrosine phosphorylation	5.17×10^{-4}	4	219
6	GO:0030335	positive regulation of cell migration	6.68×10^{-4}	5	633
7	GO:2000147	positive regulation of cell motility	8.34×10^{-4}	5	662
8	GO:0040017	positive regulation of locomotion	9.39×10^{-4}	5	678
9	GO:0051272	positive regulation of cellular component movement	9.6×10^{-4}	5	681
10	GO:0060429	epithelium development	1.39×10^{-4}	6	1532

GO biological annotations of the 3 groups of genes found in Table 5. The annotations were divided into different categories that were marked by different colors. Explanations for the colors: Yellow-response to cytokine, Violet-circulatory system and tube biology, Green-mesenchymal differentiation and cell migration.
